# Prevalence and epidemiological distribution of substance use among people living with HIV in the East African region: a meta-analysis

**DOI:** 10.3389/fpsyt.2025.1494469

**Published:** 2025-09-03

**Authors:** Hope Onohuean, Fanny Eseohe Onohuean, Charles Omara, Haneefa Saleem

**Affiliations:** ^1^ Biomolecules, Metagenomics, Endocrine, and Tropical Disease Research Group (BMETDREG), Kampala International University, Western Campus, Ishaka‑Bushenyi, Uganda; ^2^ Biopharmaceutics Unit, Department of Pharmacology and Toxicology, Kampala International University Western Campus, Ishaka-Bushenyi, Uganda; ^3^ Bloomberg School of Public Health, Johns Hopkins University, Baltimore, MD, United States

**Keywords:** prevalence, epidemiological-distribution, substance-use, HIV, East Africa, meta-analysis

## Abstract

**Background:**

The East African region lacks synthesised scientific evidence on the impact of different substances used on HIV management and treatment outcomes in this population. We meta-analysed epidemiological data on substance use among people living with HIV to determine the regional estimate of the prevalence, associated factors, and changes over time for each gender, per year or age.

**Method:**

The documents were obtained via electronic databases following Preferred Reporting Items for Systematic Reviews and Meta-Analysis (PRISMA) guidelines.

**Results:**

The 53 studies from the East African region showed a pool estimate proportion of 60.36%, 95% confidence interval (CI) (0.5301–0.6728) with an *I* ^2^ = 98.88% using the random-effects model, and *Q*-statistic (df = 52) = 4,662.95, *p* < 0.0001. The publication bias is revealed by funnel plots, 55.15%, CI (0.4637–0.6362); Egger’s linear regression test indicates *z* = 12.6415, *p* < 0.0001; and the rank correlation test of Kendall’s tau = 0.1011, *p* = 0.2955. The subgroup analysis showed an estimate of the study type: cohort, 66.10%, CI (0.5672–0.7437), *I* ^2^ = 98.60%, and cross-sectional, 58.98%, CI (0.4009–0.7555), *I* ^2^ = 99.06%. The variables of the subgroup analysis by study size indicate the following: >1,000 sample size, 76.05%, CI (0.4661–0.9203), *I* ^2^ = 99.75%, and <1,000 sample size, 62.85%, CI (0.5396–0.7095) *I* ^2^ = 100%. The meta-regression analysis of heterogeneity indicates that the covariate of countries (*R* ^2^ = 0.00%, *p* < 0.0001), types of substance use (*R* ^2^ = 0.00%, *p* < 0.0001), and study period (*R* ^2^ = 16.95%, *p* = 0.0013) significantly moderate the observed heterogeneity.

**Conclusion:**

The East African region has a high prevalence of substance use among people living with HIV, which may further increase the risk of spread of infections and signs of deteriorating physical and mental health. Comprehensive treatment and establishing interventions for substance abuse/misuse among people living with HIV could be a top health priority in the region.

## Background

HIV continues to be a serious global public health concern, having taken 40.4 million lives [32.9–51.3 million] to date and continuing to spread throughout all nations with a rising rate of new infections in many nations (1). Studies have shown that an estimated 39.0 million people live with HIV—of whom 1.8 million are children—while 19% (7.1 million) do not realise they have the virus (2–4). An estimated 25.6 million people (65.64%) globally are HIV-positive, and more than two-thirds of those individuals are young adults residing in eastern Africa, southern Africa, and other African nations (3, 4).

Over half (19.4 million, 53%) of the people living with HIV worldwide are in southern or eastern Africa in 2022 (5, 6). In Kenya, 1.6 million people are estimated to be living with HIV/AIDS, 1.1 million of whom are children (7, 8). In Uganda, approximately 1.4 million people are living with HIV/AIDS, of whom 860,000 are women and 80,000 are children (9).

The prevalence of substance abuse in East African countries is high, with a 43.70% prevalence of substance abuse coverage among men in the region (10). The Global Burden of Disease (GBD) data indicate that the eastern region of sub-Saharan Africa has the highest age-standardised prevalence of alcohol, cannabis, and other substance use, highlighting the severity of the issue in this area (11). Moreover, the existence of many transit port services in the Middle East Gulf States has contributed to the smuggling of substance abuse drugs in the region, further exacerbating the problem (12–14).

HIV and substance use are two interconnected public health issues that have significant impacts on individuals, families, and communities (15, 16). Understanding the prevalence and epidemiological distribution of substance use among people living with HIV in the East African region is crucial for effective prevention, intervention, and treatment strategies. In the East African region, substance abuse is a significant issue among people living with HIV, with a prevalence ranging from 7% to 16%, indicating a substantial proportion of individuals who either abuse or are dependent on alcohol and other substances (17, 18). Furthermore, this co-occurrence of substance use and HIV has been shown to negatively impact medication adherence, leading to potential complications in treatment outcomes (14, 19–22). Moreover, there is evidence that alcohol use is explicitly related to more significant HIV transmission risks, highlighting the urgent need for interventions targeting substance use in this population (23–25).

Evidence shows that substance abuse significantly impacts adherence to anti-retroviral therapy (ART) (17, 26). Specifically, anxiety disorders, depression, and disorders associated with substance abuse were found to be important factors affecting adherence. The substances frequently abused among people living with HIV in Uganda are alcohol, cannabis, khat, tobacco, marijuana, or other illicit drugs (cocaine, glue, and heroin), while alcohol and marijuana have the highest prevalence (27, 28); in Kenya, either licit (legal) substances like alcohol (beer, wines, and spirits), tobacco, and khat (miraa) or illicit (illegal) substances like heroin, cocaine, local brew (chang’aa), bhang, kuber, and mandrax are abused (29, 30); in Tanzania, approximately 50% of the population living with HIV/AIDS are reported to abuse or have used drug or alcohol disorders while the most prevalent substance use includes injection of heroin, methamphetamine, cocaine, Diclopa and valium, marijuana, or heroin combined with marijuana called kokteli (translated to “cocktail”) (31).

Therefore, it is essential to further investigate the relationship between substance use and HIV in East Africa using scientific evidence-based data, thereby identifying the specific substances commonly abused and their impact on HIV transmission, treatment outcomes, and overall public health. This knowledge would not only contribute to the development of tailored interventions and prevention strategies but also promote better integration of substance abuse treatment into HIV care services. Understanding the prevalence and epidemiological distribution of substance use among people living with HIV in East Africa is crucial for effective prevention, intervention, and treatment strategies. However, there is a lack of comprehensive data on this topic, as previous studies have focused mostly on HIV/AIDS or HCV infection and did not examine the specific prevalence of substance abuse among individuals living with HIV (4, 32–34). Additionally, there is a lack of research exploring the impact of different substances on HIV transmission and treatment outcomes in this population. Utilising a meta-analysis of existing studies on this topic, we can gather comprehensive scientific data that can inform policy and program development to address the dual challenges of HIV and substance abuse in East Africa. In addition, a meta-analysis of existing studies on substance use among people living with HIV in East Africa can address these gaps in knowledge and provide valuable insights for public health planning and interventions. Therefore, we analyse epidemiological data of existing studies on substance use among people living with HIV to determine the East African regional estimate prevalence of substance use among people living with HIV and to examine epidemiological factors, such as East African nations (countries), the types of substance use among people living with HIV, study design, sample size, and study period, that may contribute to heterogeneity. Thus, there are indications that the prevalence of substance use may have changed over time for each gender, per year or age.

## Methodology

### Search strategy

In this study, we conducted a literature search in electronic databases such as Web of Science (WOS), Scopus, and PubMed, and article references in accordance with the standard Preferred Reporting Items for Systematic Reviews and Meta-Analysis (PRISMA) (34–36). The Boolean keywords “Epidemiology AND HIV OR Human immunodeficiency virus OR HIV (Aids) AND Substance use OR Alcohol OR Narcotics OR Nicotine OR Marijuana OR Heroin OR Cocaine OR Methamphetamine) AND East Africa AND (Tanzania OR Kenya OR Uganda OR Rwanda OR Burundi OR South Sudan” that took the form of title words or headings for medical subjects were used to retrieve studies published between 1988 and 7 December 2022, and later updated on 27 January 2023 at approximately 1.23 GMT+2.

### Study selection criteria

Articles that reported substance use as the cause of behavioral changes or neurological disorders, especially illegal substances, such as alcohol, khat, marijuana, heroin, cocaine, methamphetamine, drugs, or injected drugs, among people living with HIV in the East African region were included. All articles that reported substance use identified by ICD-9-CM, ICD-10-CM, and ICD-10 T-codes, as determined by the Substance Abuse and Mental Health Services Administration (SAMHSA), were included. However, articles were restricted to the English language only. Review articles, editorials, early access papers, proceeding papers, notes, short surveys, letters to editor, and abstracts were excluded.

### Outcomes of interest

The outcome of interest includes prevalence of substance use (or specific correlates, e.g., age, gender, socioeconomic status, and national/subnational community) among people living with HIV in the East African region.

### Data extraction and outcomes of interest

The performance indicators for this meta-analysis were the first authors’ names, the year of publication, the total population, the number of positive instances (prevalence of substance use), the nation where the study was conducted, the examined source, the study period, and the study type adopted from our previous studies (35, 36). These indices were extracted from the qualified articles' findings, discussions, figures, and tables mined as metadata by two groups of investigators (H.O. and C.O, and F.E.O. and S.H.) independently. Additionally, the proficiencies and discrepancies were assessed/discussed until an agreement between the leaders of both groups of investigators (H.O. and S.H.) is reached. Then, documentation concerning the homogeneity, consistency, and heterogeneity between the study populations was performed, and further statistical analysis was based on the investigators’ predetermined criteria for the study.

### Assessment of data quality

The Newcastle–Ottawa Scale (NOS), authorised by the Agency for Healthcare Research and Quality (AHRQ) (https://www.ohri.ca/programs/clinical_epidemiology/oxford.asp), was used to evaluate the data quality for this meta-analysis. Three criteria—study group selection, group comparability, and outcome measurement—were used to rate the studies’ quality; these categories were graded using a star system.

### Statistical analysis

Among the 53 included studies, the Wilson method of confidence intervals (CIs) was used to compute raw proportions and 95% CIs. The weighted overall effect size (weighted average proportion) was determined for the original study random-effects meta-analysis based on the individual effect sizes and related sample variances. Using the argument method=“DL”, for restricted maximum-likelihood estimator. The logit transformation was employed to produce the pooled prevalence in order to improve the statistical characteristics because the proportion between studies varies from 0.005 to 1 (37). Meta-regression analyses were used to quantify the impact of the heterogeneity and homogeneity of the studied populations. A forest plot was created after the epidemiological distribution subgroup analysis. Egger’s test for asymmetry was used to generate funnel plots that compare publication bias. The significance of the bias was then assessed using the rank correlations test and Kendall’s model. Each analysis was two-tailed with a 0.05 significance threshold and carried out using the statistical program R 4.0.5 (35, 38–40).

## Results

### Literature search summary

### Quality assessment

**Supplementary Table 1** shows the assessment questions in each article’s respective domain, and the quality evaluation scores of the included articles were marked by a star (*). Because of the lack of comparison studies in the publications that were included, the NOS comparability variables were rated zero stars by all of the meta-synthesised studies. The quality rankings for the remaining studies vary from four to seven. Of the possible eight points, 38 studies scored seven points, 17 scored six points, and 1 scored five points.

### Characteristics of the included studies

**Figure 1:**
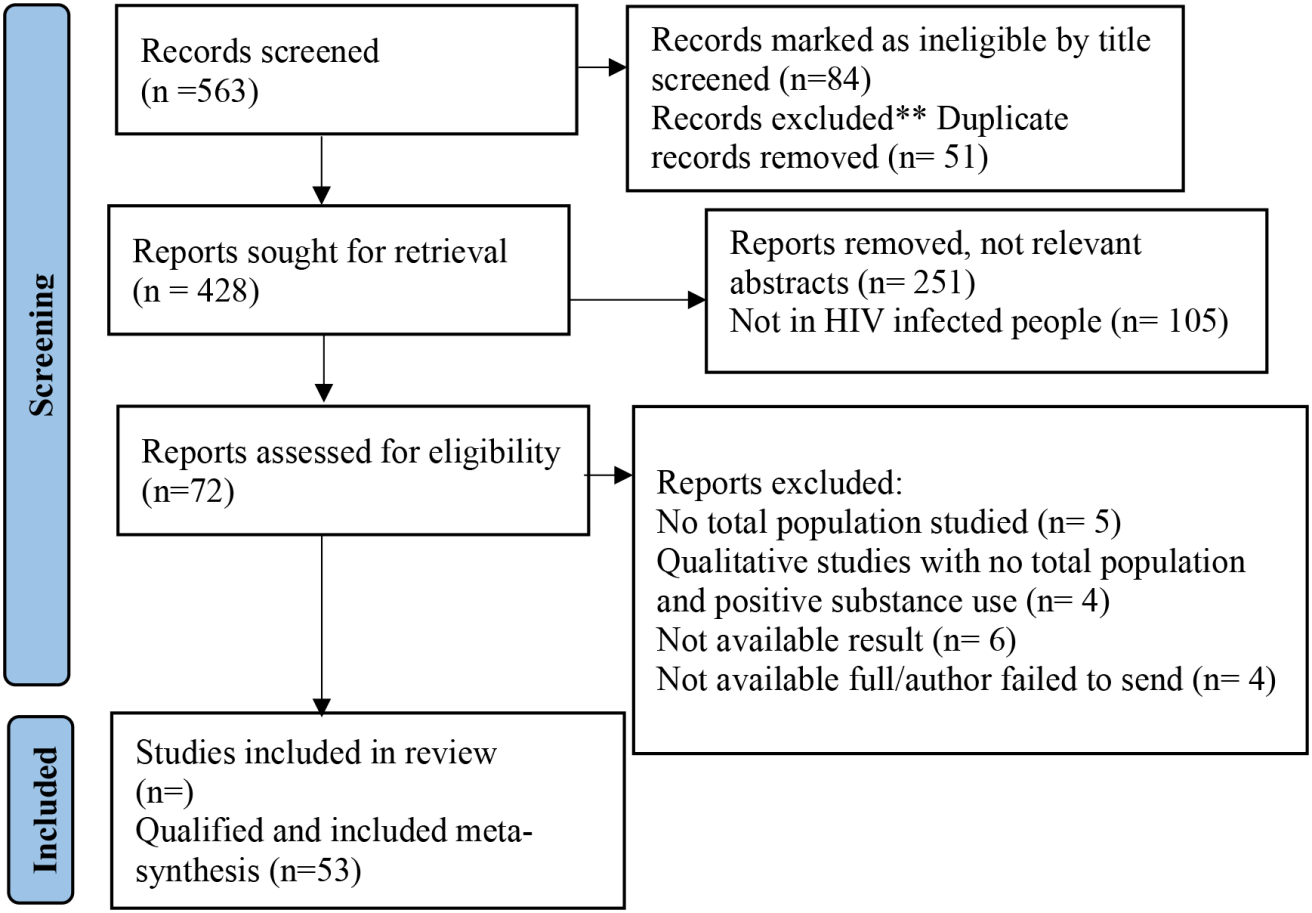
Flowchart of PRISMA guidelines for the study selection and meta-analysis.

Based on the search term on the epidemiological data of substance use among people living with HIV in the East African region, we identified a total of 618 records from WOS (*n* = 225, 36.4%), Scopus (*n* = 69, 11.2%), and PubMed (*n* = 324, 54.4%). Initial screening of the records from the databases (excluding reviews, notes, editorials, abstracts, etc.) yielded *n* =563 records (91.1%). Further exclusion of ineligible (84) and duplicate articles (51) resulted in *n* = 428 records (69.3%). Further review of the abstracts of the reported documents resulted in *n* = 72 (11.7%) eligible articles, and 53 studies were included in the meta-analysis detailed in **Figure 1**.

From the sample-tested population of 38,401 in the East African region, 16,622 (43.3%) used different substances among people living with HIV. The population size of the article metadata ranges from 51 to 14,199 within the study period of 2 months to 4 years, as reported by the authors. The study types of most of the articles were cross-sectional surveys, cohort studies, or prospective cohort studies (**Table 1**), and the doi or title to access the included studies on the East Africa regional prevalence of substance use among people living with HIV are presented in **Supplementary Table 2**. The East African nations reporting on substance use among people living with HIV include Ethiopia (*n* = 7), Kenya (*n* = 16), Tanzania (*n* = 13), and Tanzania and others (*n* = 2). Chang et al. (48) report on studies from Eswatini, Malawi, Namibia, Tanzania, Zambia, and Zimbabwe, and Medley et al. (74) report on studies from Tanzania, Kenya, Namibia, and Uganda (*n* = 15 articles) depicted in the **Supplementary Figure 1**. Indicating the selected studies hotspot on the East Africa regional prevalence of substance use among people living with HIV. However, 11 authors report the positive cases in percentages (%), which were converted to actual numbers.

**Table 1:** Summary characteristics of the inclusive studies on the East Africa regional prevalence of substance use among people living with HIV.

Authors	Country/region	Population size	Substance use	Study period	Study type
27	Uganda	479	Marijuana, alcohol	Nil	Cross-sectional survey
22	Uganda	1,134	Alcohol	12 months	Cross-sectional survey
41	Kenya	614	Alcohol	9 months	Randomised clinical trial
42	Tanzania	611	Alcohol	October and December 2017	Cross-sectional survey
25	Uganda	59	Alcohol	12 months	Cross-sectional survey
21	Uganda	300	Tonto and waragi	2016–2017	Cross-sectional survey
43	Tanzania	53	Opioid	Nil	Cross-sectional survey
44	Tanzania	136	Methadone	October 2015 and May 2017	Cross-sectional survey
45	Tanzania	812	Alcohol	From March 2012 until April 2013	Cross-sectional survey
20	Ethiopia	195	Alcohol, cigarette smoking	Nil	Cross-sectional survey
46	Uganda	256	Alcohol	Nil	Cross-sectional survey
47	Uganda	445	Alcohol	January and April 2018	Cross-sectional survey
48	Eswatini, Malawi, Namibia, Tanzania, Zambia, and Zimbabwe	14,199	Alcohol	2015–2017	Cohort
49	Uganda	408	Alcohol	Nil	Randomised controlled trials
29	Kenya	464	Alcohol	Between November 2018 and September 2019	Cross-sectional survey
50	Uganda	421	Alcohol	From May 2018 through March 2020	Cohort
51	Kenya	366	Alcohol	October 2012 and March 2018.	A prospective cohort study
52	Kenya	451	Injected drugs	July 2012–February 2013	Cross-sectional survey
53	Ethiopia	237	Heroin, marijuana, khat	Between 26 March and 22 May 2015	Cross-sectional survey
54	Uganda	446	Alcohol	September 2011 to August 2014	Prospective cohort
55	Ethiopia	527	Alcohol	May to June 2015	Cross-sectional survey
56	Uganda	751	Alcohol	Nil	Cohort
57	Ethiopia	322	Khat	Nil	Cross-sectional survey
58	Ethiopia	322	Alcohol, khat	2013–2014	Cross-sectional survey
59	Tanzania	620	Injected drugs	2015–2017	Cross-sectional survey
60	Uganda	325	Alcohol	Nil	Cross-sectional survey
61	Kenya	118	Injected drugs	December 2012–January 2014	Longitudinal qualitative analysis
62	Uganda	1027	Alcohol	April 2008–April 2009	Cohort study
63	Kenya	112	Alcohol	March and September 2014	Cross-sectional survey
64	Kenya	60	Alcohol	Nil	Cross-sectional survey
65	Kenya	1,185	Heroin	May and December 2012	Cross-sectional survey
66	Kenya	118	Heroin	December 2012/January 2013	Qualitative study
67	Kenya	109	Methadone	2014–2015	Qualitative longitudinal study
68	Kenya	352	Injected drugs, e.g., heroin, khat, and marijuana	January and March 2011	Cross-sectional survey
69	Kenya	151	Alcohol, marijuana, prescription drugs	October 2013–July 2014	Quantitative survey
70	Kenya	186	Heroin	Nil	Cross-sectional survey
31	Tanzania	480	Heroin	June and August 2014	Cross-sectional survey
71	Ethiopia	398	Khat	September 2012–2015	Cross-sectional survey
72	Tanzania	629	Methadone	February 2011 to January 2013	Cross-sectional survey
73	Uganda	59	Alcohol	Nil	Prospective cohort study
74	Tanzania, Kenya, and Namibia	3,538	Alcohol	October 2009 and April 2010	Cross-sectional survey
75	Ethiopia	389	Alcohol	2012–2014	Cross-sectional survey
76	Uganda	496	Tobacco	Nil	Prospective cohort study
77	Kenya	58	Injected drugs	February and March 2010	Cross-sectional survey
78	Uganda	329	Alcohol	April and October 2006	Cross-sectional survey
79	Tanzania	509	Heroin	2007–2010	Cross-sectional survey
80	Kenya	297	Alcohol, khat, marijuana, heroin, cocaine, glue, or petrol	July 2007–2010	Cross-sectional survey
81	Tanzania	537	Injected drugs	May 2005 and September 2006	Cross-sectional survey
82	Tanzania	1,050	Alcohol	2002 and 2003	Prospective cohort study
83	Tanzania	537	Heroin, marijuana, alcohol	2005–2006	Cross-sectional survey
84	Tanzania	374	Heroin	October 2003 and January 2004	Cross-sectional survey
85	Tanzania	51	Heroin	February and July 2003	Cross-sectional survey
86	Kenya	299	Alcohol	Nil	Cross-sectional survey

Not reported = nil.

### Epidemiological distribution of substance use among people living with HIV in the East African region

**Figure 2:**
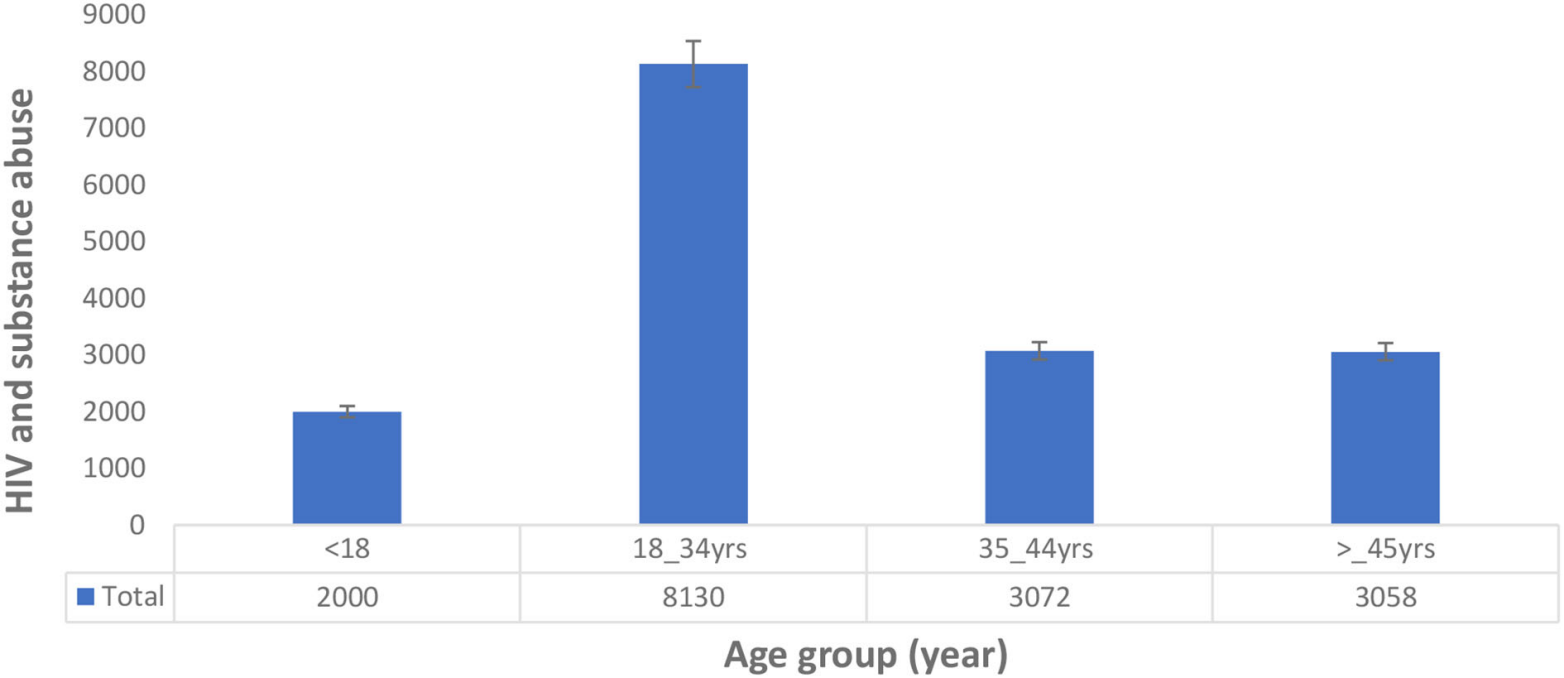
Age distribution of substance use among people living with HIV in the East African region.

**Figure 3:**
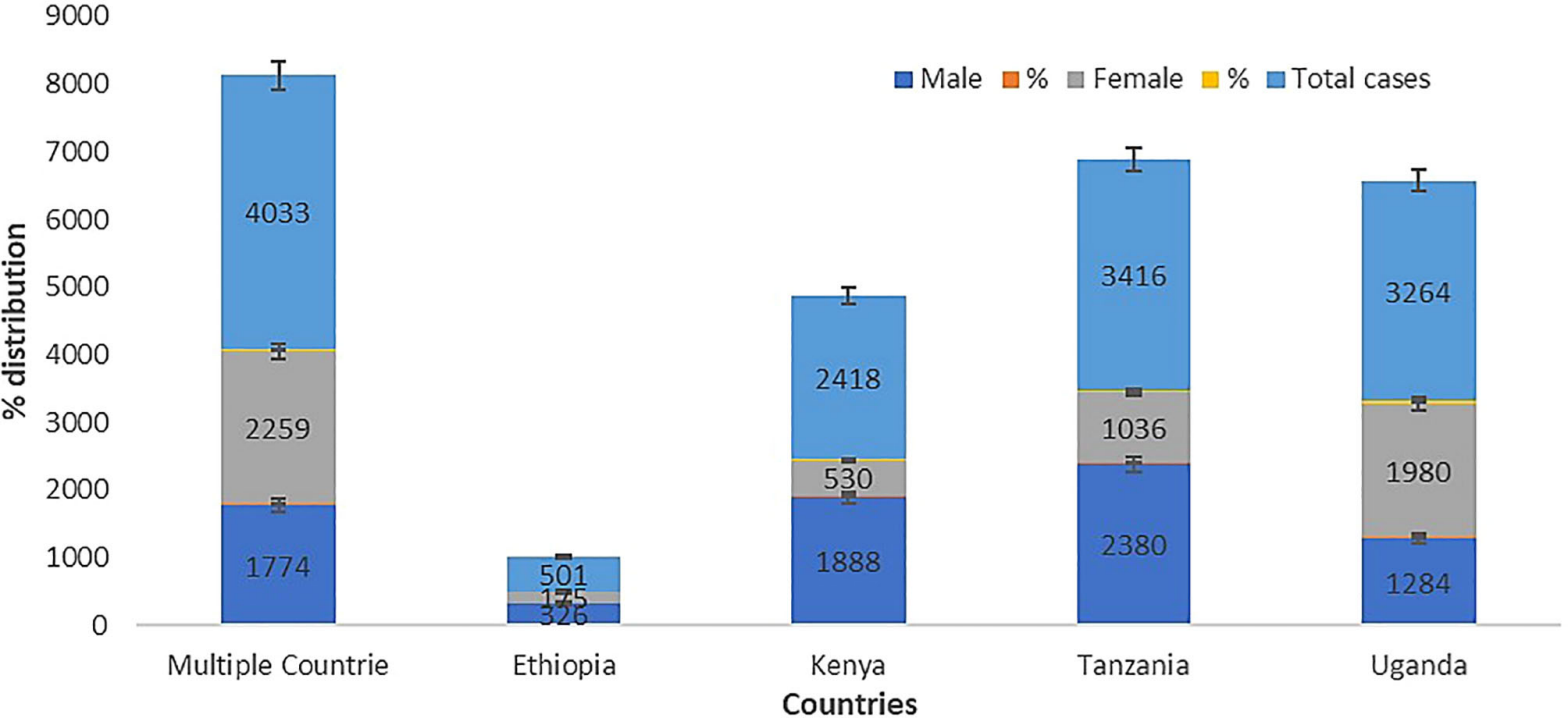
Gender distribution of substance use among people living with HIV in the East African region.

We analysed the age, gender, and types of substance use that may help to understand and identify patterns of geographic evolving trends on the epidemiological characteristics of substance use among people living with HIV in the East African region. The results show that 49 studies (92.5%) reported either the average age, mean age, median age, or group age of the participants, while only 4 studies (7.5%) did not report the age of their participants. Furthermore, our meta-synthesis reveals that the 18 to 34 years and 35 to 44 years age groups have the highest prevalence of substance use among people living with HIV in the East African region (**Figure 2**). In addition, the prevalence of substance use among people living with HIV was higher in women in Uganda and multiple countries, while in Kenya and Tanzania, prevalence was the highest in men, as detailed in **Figure 3**.

**Figure 4:**
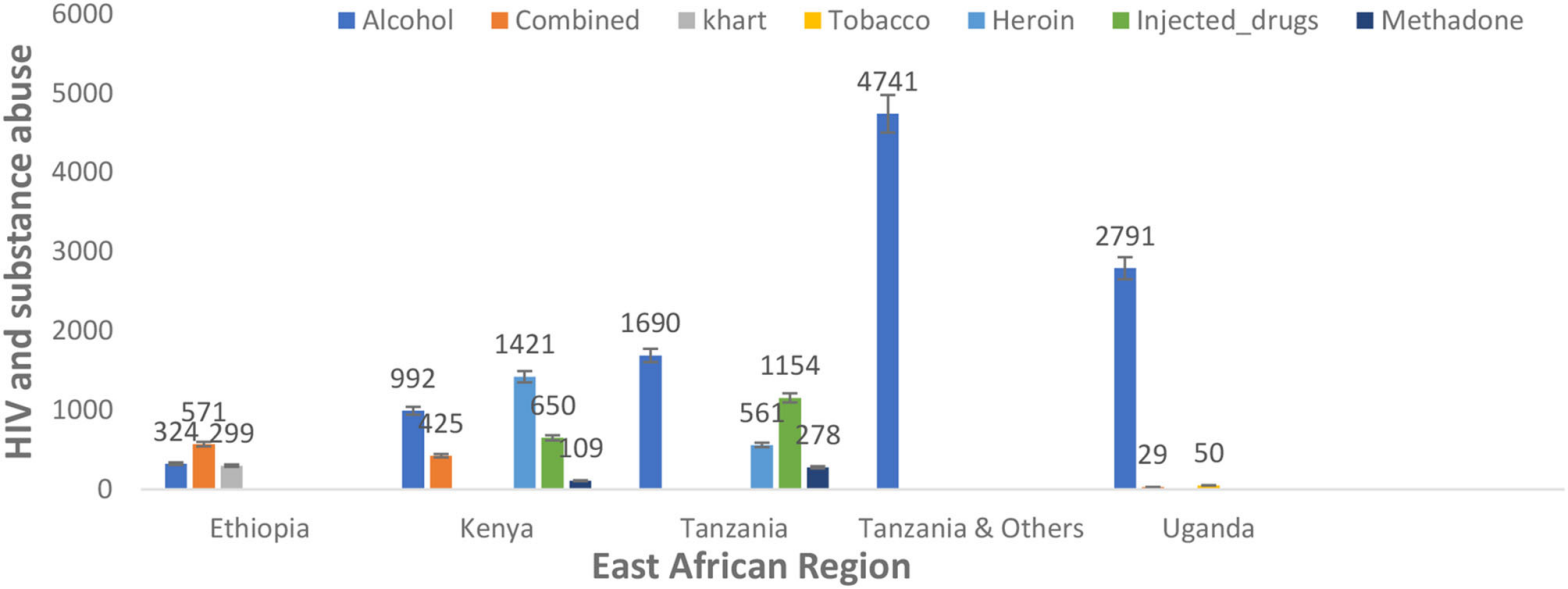
Type of substance use among people living with HIV in the East African region.

In addition, there was variation in the type of substance use among people living with HIV in the different reported nations in the East African region. However, alcohol was commonly used among people living with HIV in Ethiopia (324, 1.95%), Kenya (992, 5.97%), Tanzania (1,690, 10.17%), Tanzania and others (4,741, 28.52%), and Uganda (2,791, 16.79%). Heroin (1,421, 8.549% and 561, 3.375%) and injected drugs (650, 3.91% and 1,154, 6.94%) were prevalent in the population of Kenya and Tanzania, respectively. Combined substance use (571, 3.44%) was prevalent in the Ethiopian population, as detailed in **Figure 4**.

### The prevalence estimates of substance use among people living with HIV in the East African region

**Figure 5:**
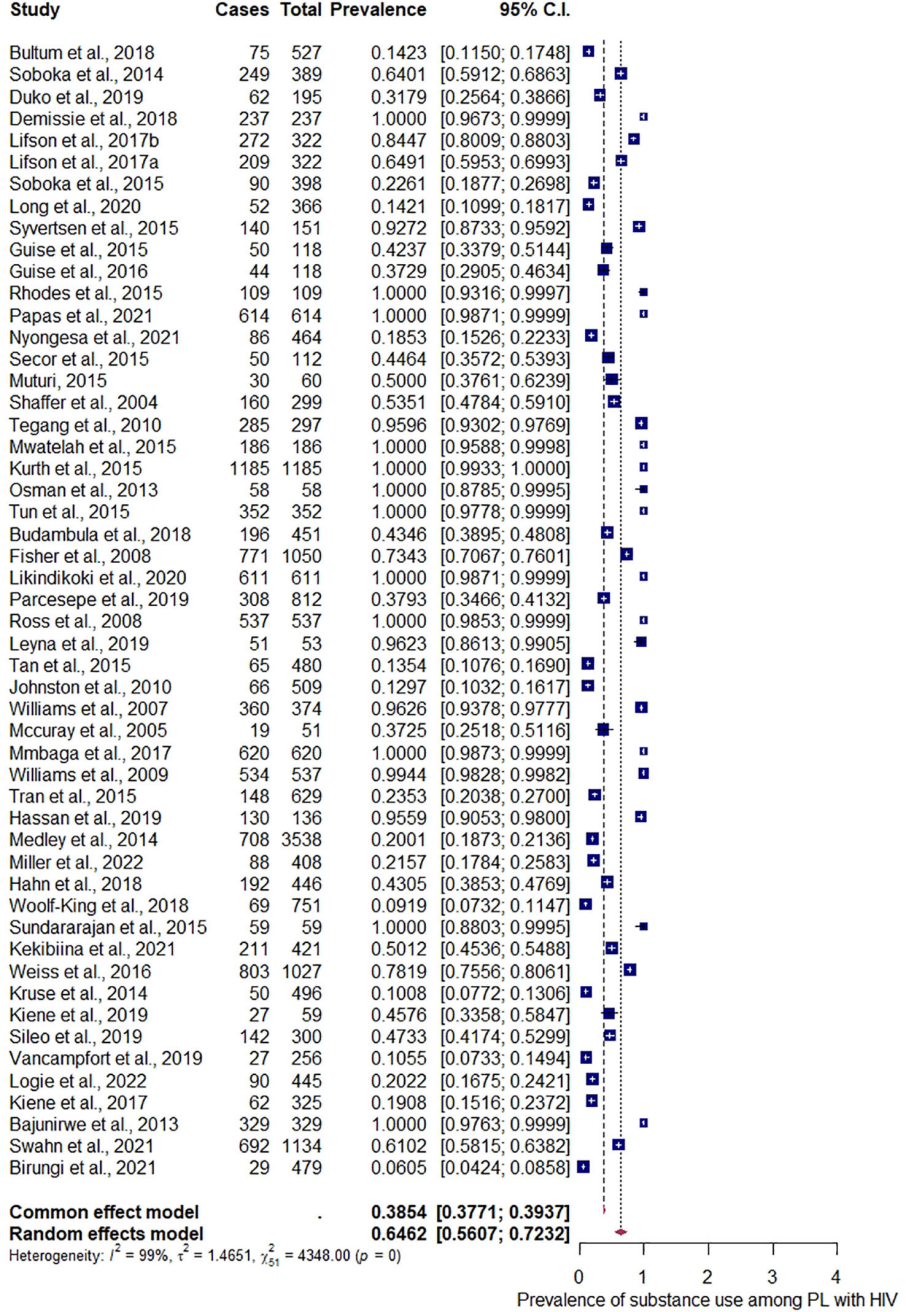
Forest plot for the prevalence of substance use among people living with HIV.

**Figure 6:**
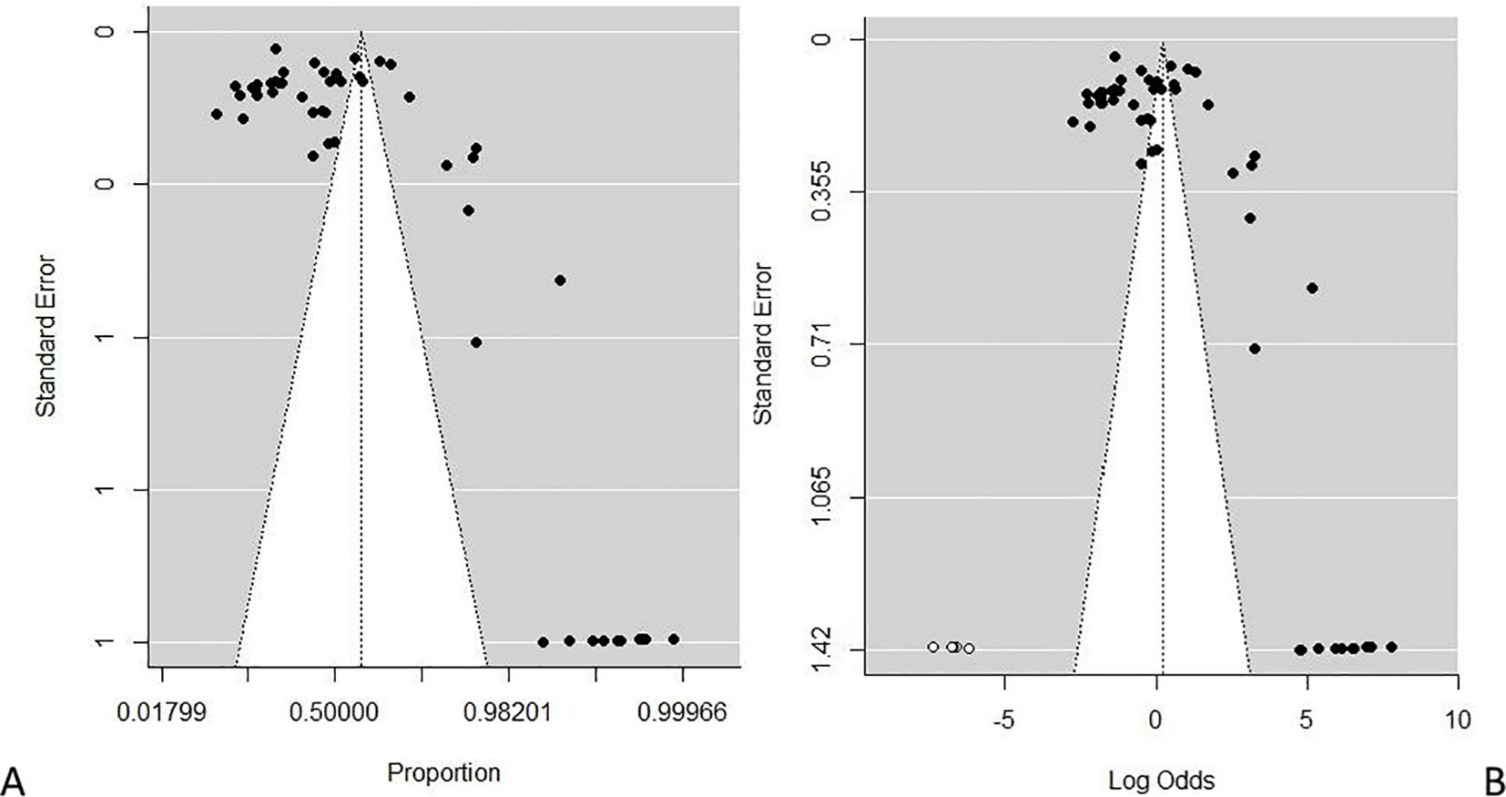
**(A, B)** Plots of publication bias testing for the prevalence of substance use among people living with HIV. **(A)** Funnel proportion plots. **(B)** Funnel trim fill log odds plot.

During the meta-analysis, one outlier (48) was identified and removed from the analyses; the overall pooled estimate of substance use among people living with HIV according to epidemiological studies in the East African region shows a proportion of 60.36%, 95% CI (0.5301–0.6728), with *I* ^2^ = 98.88% using the random-effects model, and a significant *Q* statistic (df = 52) of 4,662.95, *p* < 0.0001. The result suggests a high proportion among studies, and a significant variation depicts that the allotted studies do not share a common effect size. Furthermore, the meta-analysis contains substantial heterogeneity (**Figure 5**). The publication bias evaluation through a funnel plot shows 55.15%, CI (0.4637–0.6362). Funnel plots were used to determine the publication bias. Each point denotes a separate study on the designated association. The vertical line denotes the mean effect size. However, the points are dispersed asymmetrically, which shows publication bias (**Figures 6A, B**). The Egger test model (*p* < 0.0001) indicates a significant level of publication bias. Egger’s linear regression test indicates *z* = 12.6415, *p* < 0.0001 and the rank correlation test of Kendall’s tau = 0.1011, *p* = 0.2955.

### Source of heterogeneity analysis for the regional prevalence of substance use among people living with HIV: meta-regression

The likely sources of heterogeneity were determined in five certain factors covariates by univariate meta-regression analyses. These includes countries: Ethiopia, Kenya, Tanzania, Uganda, and other multiple/mixed nations studies such as (Eswatini, Malawi, Namibia, Tanzania, Zambia and Zimbabwe); substance use: alcohol, heroin, methadone, injected drugs, combined (alcohol, cigarette smoking, heroin, marijuana, khat) tobacco; study design: cross-sectional or cohort; study sample size: More than 1000 or less than 1000; study period: years, months and none reported. The moderator effect accounted for and the contribution of each covariate estimate were determined by *R* ^2^ (amount of heterogeneity accounted for) and *p*-values. The results of the included studies showed that the study design (*R* ^2^ = 1.08%, *p* = 0.2787) and study sample size (*R* ^2^ = 0.00%, *p* = 0.4276) were not significantly associated with the prevalence of substance use among people living with HIV in the study region. However, the covariate of countries (*R* ^2^ = 0.00%, *p* < 0.0001), types of substance use (*R* ^2^ = 0.00%, *p* < 0.0001), and study period (*R* ^2^ = 16.95%, *p* = 0.0013) significantly moderate the observed heterogeneity. Moreover, the following multivariate mixed effects meta-regression model was developed for the study (countries, type of substance use, and period), considering the aforementioned variables significantly influencing the overall prevalence heterogeneity. These three covariates accounted for 48.41% of the heterogeneity in the ND prevalence estimate (*R* ^2^ countries + types of substance use + period = 0.00%, *p* countries + types of substance use + period < 0.001).

### Variations in the regional prevalence of substance use among people living with HIV: subgroup analysis

**Figure 7:**
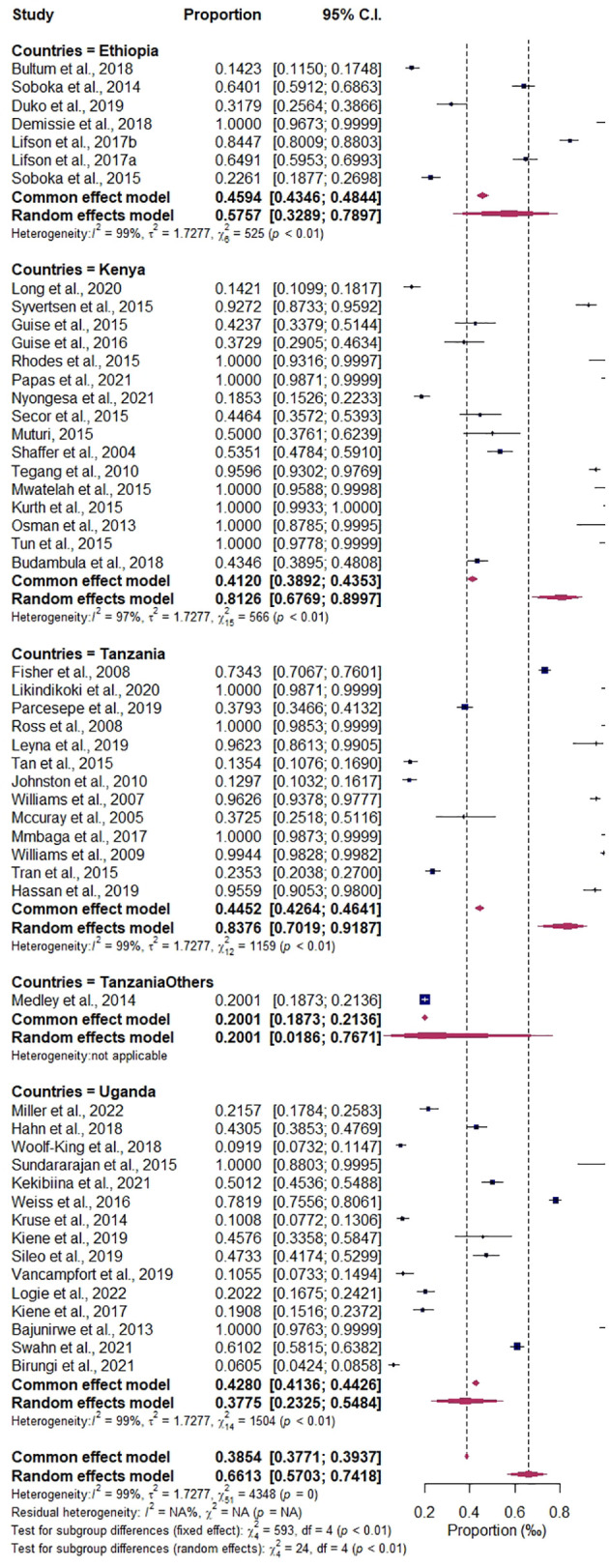
Forest plot of the subgroup analysis by countries in the region.

**Figure 8:**
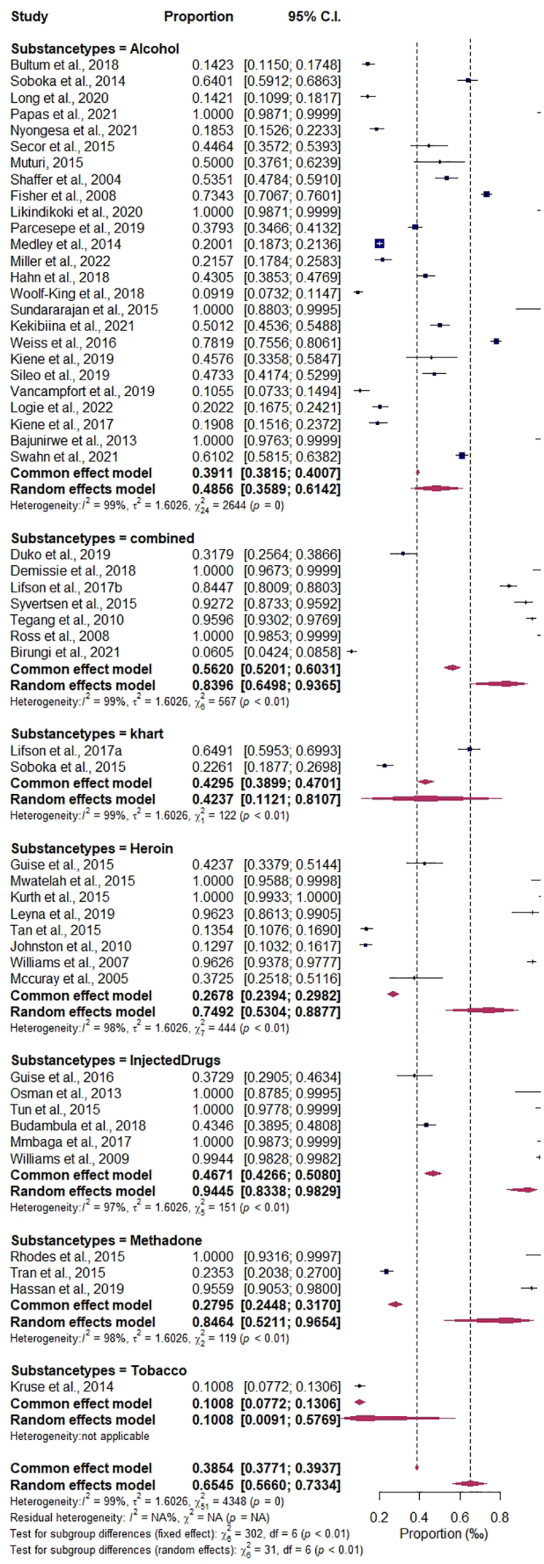
Forest plot of the subgroup analysis by type of substance use.

**Figure 9:**
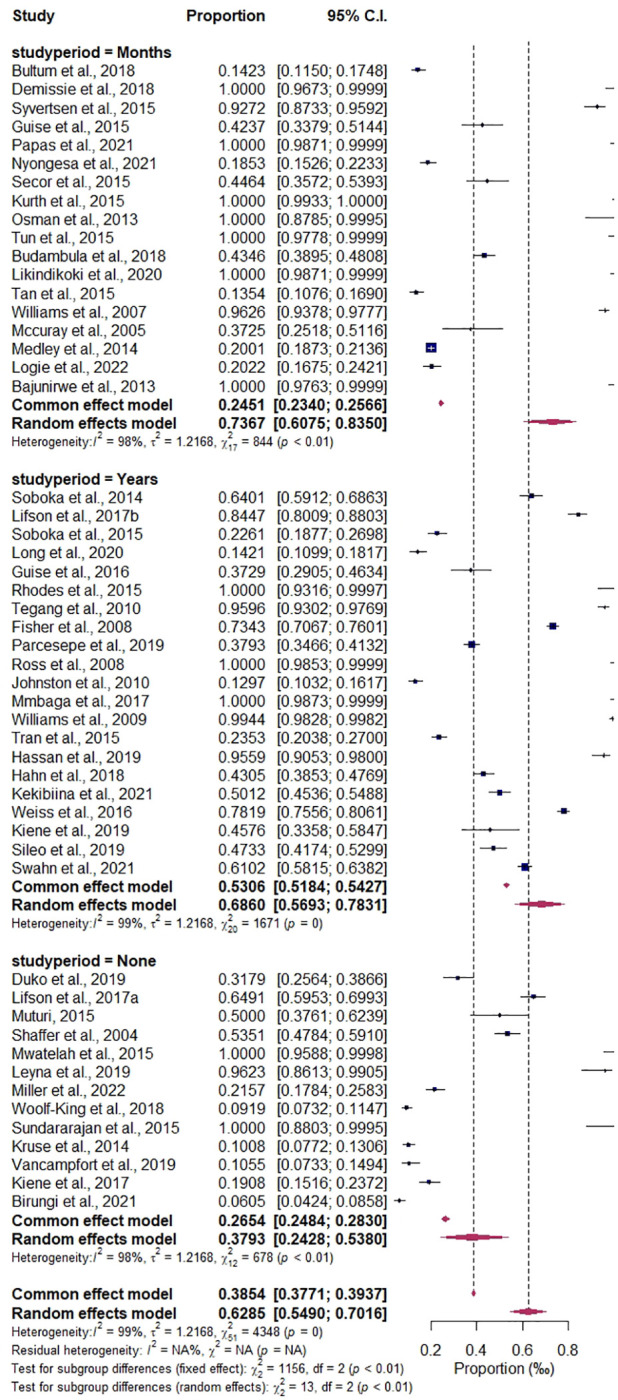
Forest plot of the subgroup analysis by study period.

The categorical variables of study design (cohort or cross- sectional), show the probable variations to be highest prevalence estimate in cohort 66.10%, CI (0.5672–0.7437), I2 = 98.60%, compared to the cross-sectional group of 58.98%, CI (0.4009–0.7555), I2 = 99.06%. The variables of the subgroup analysis by study size were the highest in the above 1,000 sample size, 76.05%, CI (0.4661–0.9203), *I* ^2^ = 99.75%, compared to those in the less than 1,000 sample size, 62.85%, CI (0.5396–0.7095); details are found in **Supplementary Figures 2** and **3**. Among the countries subgroups variations that were significantly associated with the prevalence of substance use among people living with HIV are: Kenya, 80.23%, CI (0.6702–0.8902), *I* ^2^ = 97.35%; Tanzania, 85.23%, CI (0.7051–0.9330), *I* ^2^ = 98.97%; Ethiopia, 57.32%, CI (0.3314–0.7845), *I* ^2^ = 98.86%; Tanzania and others, 20.01%, CI (0.1873–0.2136), *I* ^2^ = 0.00%; Uganda, 37.21%, CI (0.2346–0.5339), *I* ^2^ = 99.07%, as detailed in **Figure 7**. The prevalence estimates of different types of substance use are as follows: alcohol, 46.75%, CI (0.3564–0.5819), *I* ^2^ = 99.09%; methadone, 90.44%, CI (0.1945–0.9973), *I* ^2^ = 98.32%; heroin, 80.71%, CI (0.5147–0.9429), *I* ^2^ = 98.42%; injected drugs, 95.94%, CI (0.8362–0.9909), *I* ^2^ = 96.68%; khat, 42.37%, CI (0.1076–0.8177), *I* ^2^ = 99.18%; tobacco, 10.08%, CI (0.0772–0.1306), *I* ^2^ = 0.00%; and combined drugs, 89.09%, CI (0.5681–0.9807), *I* ^2^ = 98.94%; details are presented in **Figure 8**. The study period subgroup analysis shows the following group prevalence estimates: months, 73.38%, CI (0.6053–0.8321), *I* ^2^ = 97.99%; years, 68.18%, CI (0.5686–0.7769), *I* ^2^ = 98.80%; and none (articles that do not report their study periods), 39.65%, CI (0.2421–0.5747), *I* ^2^ = 98.23%, as depicted in **Figure 9**.

Prevalence and epidemiological characteristics are significant among the subcategories of specific substance use types and nations among people living with HIV in the East African region. The result shows alcohol to be the mostly used substance in the studied population, in comparison to the following: combined substances [**p = 0.001, 95% CI (64.98–93.65)], heroin [*p = 0.01, 95% CI (53.04–88.77)], injected drugs [***p < 0.001, 95% CI (83.38–98.29)], and methadone [**p = 0.01, 95% CI (52.11–96.54)], indicating a significant impact on public health and HIV prevention and management in the region. On the other hand, Kenya [^a^ *p* = 0.05 (67.69–89.97)] and Tanzania [*p = 0.01 (70.19–91.87)] have been significantly impacted by substance use among people living with HIV compared to the population of Ethiopia in the reported studies detailed in **Table 2**.

**Table 2:** Pooled estimates of types of substance use and East African regional epidemiological characteristics.

				Random-effects model		
Variables	Studies	Estimate effect	95% CI	Prevalence (%)	95% CI (%)	Heterogeneity (*I* ^2^)
Types of substance use						
Alcohol	25	−0.0576	−0.5804–0.4856	48.56	35.89–61.42	99%
Combined **	7	1.7126	0.5515–0.8396	83.96	64.98–93.65	99%
Heroin *	8	1.1521	0.0479–0.7492	74.92	53.04–88.77	98%
Injected drugs ***	6	2.8913	1.5633–0.9445	94.45	83.38–98.29	97%
Khat	2	−0.2499	−2.0879–0.4237	42.37	11.21–81.07	99%
Methadone *	3	1.7639	0.0598–0.8464	84.64	52.11–96.54	98%
Tobacco		−2.1307	−4.6831–0.1008	10.08	0.91–57.69	–
Significance codes: 0 “***” 0.001 “**” 0.01 “*” vs. alcohol.						
East African nations						
Ethiopia	7	0.305	−0.713–0.5757	57.57	32.89–78.97	99%
Kenya ^a^	16	1.1619	−0.0892–0.8126	81.26	67.69–89.97	97%
Tanzania*	13	1.3358	0.0506–0.8376	83.76	70.19–91.87	99%
Tanzania and other nations	1	−1.6906	−4.4618–0.2001	20.01	1.86–76.71	–
Uganda	15	−0.8051	−2.0372–0.3775	37.75	23.25–54.84	99%
Significance codes: 0.01 “*” 0.05 “^a^” vs. Ethiopia.						

## Discussion

### Evidence of epidemiological data utilisation for substance use prevention/management strategies among people living with HIV

Illicit substance use is a global public health issue and a major risk factor for spreading HIV/AIDS, especially in low- and middle-income Asian countries (72). The effective utilisation of epidemiological scientific evidence is essential for developing and implementing evidence-based substance abuse prevention and management strategies among people living with HIV, as it will enable a targeted, informed, and responsive approach to address the complex interplay between HIV and substance use.

The prevalence and epidemiological distribution of substance use among people living with HIV in the East African region using combined scientific data have not been documented to understand the patterns, trends, and impact on HIV prevention and management. We, therefore, believe that this first scientific evidence will suggest the current substance use and its association with increasing HIV transmission risks for public health engagement. Furthermore, our epidemiological evaluation of substance use among people living with HIV in the East African region provides valuable insights into the prevalence, risk factors, and consequences of substance use within this population, helping to tailor interventions to address specific needs.

Overall, the obtained high pool estimate proportion of 60.36% with significant heterogeneity in our findings indicates that substance use is a serious public health concern for people living with HIV in the East African region. The included studies show significant variability in substance use among people living with HIV in the region, which is consistent with other researchers’ findings (27, 48, 54, 69, 74, 80). Our findings are significantly higher than the documented prevalence of substance use in the different populations of people living with HIV in Africa, such as alcohol, tobacco, cannabis, and other drugs (11, 87, 88), and that in Australia, Europe, and USA (89, 90). The prevalence of alcohol use disorder (AUD) among people living with HIV/AIDS in Africa was estimated to be approximately 22% (88). The epidemiologic evidence available varied depending on the study’s origin, location, methodologic quality, method utilised, sample size, or sample period and duration, which could explain some heterogeneity. Sample size differences across studies can significantly impact the findings. Larger studies generally provide more precise estimates and may dominate the analysis if not properly accounted for. On the other hand, smaller studies often have higher variability and can introduce bias or overstate the effect size due to their lower precision. However, we engaged the random-effects models’ weighting techniques to account for variations between studies, including sample size differences, by assuming that there is heterogeneity in the true effect across studies. This helps in reducing the influence of sample size disparities while still considering them. However, our analysis shows that the study design and study sample size do not significantly contribute to or are associated with the prevalence of substance use among people living with HIV in the study region. However, this variance may arise from the fact that individuals with terminal illnesses, like HIV, may turn to alcohol as a coping mechanism to manage the psychological pain brought on by the severity of their sickness and the side effects of anti-retroviral medications. Therefore, the high prevalence in the region, as identified by this analysis, is a call for further findings to understand the scope of the issue, enabling the development of targeted interventions.

### Demographic delineation and impact on HIV/AIDS prevention and management in the East African region

Our meta-synthesis reveals a high prevalence of substance use among people living with HIV in the age group of 18 to 44 years in the East African region, which is similar to other studies that showed substance use among people living with HIV to be high in older people (50, 51, 91). There are generally discrepancies in the gender prevalence of substance use among people living with HIV. However, our analysis shows a high prevalence of substance use among women in the Uganda population and other countries studied, while in Kenya and Tanzania, there is a high prevalence of substance use among men. Shokoohi et al. (92) indicated that women living with HIV have high rates of cigarette smoking, cannabis usage, crack/cocaine use, and heroin use compared to the general population, and HIV-positive women are more prone to inject drugs (93). Lancaster et al. (94) and Weiss et al. (62) previously suggested that the high rate of alcohol use in Kenyan women and female sex workers in Malawi may be due to a lack of HIV infection awareness among the HIV-infected group.

Additionally, the disparity in gender prevalence may result from differences in sociocultural facets of an individual’s life; for instance, some cultures may forbid women from consuming alcohol, and many environmental factors may also contribute to this discrepancy. Studies have suggested that high-risk behaviours, work environments, unsafe sexual practices, and obstacles to using health services are some of the factors that lead to substance use among African women living with HIV (30, 48, 62, 88). Therefore, healthcare accessibility, depression, and programs focused on women-centred harm reduction could be of utmost benefit for substance addiction among women living with HIV in the East African region.

### Conglomeration characteristics of East Africa regional estimates and their contributions to substance use among people living with HIV, implication for public health

The meta-regression analysis covariates of countries, types of substance use, and study period significantly moderate the observed heterogeneity, whereas the multivariate mixed-effects meta-regression model also influences the overall prevalence heterogeneity. This implies that the level or rate of substance use in the region’s different nations varies significantly. Moreover, the subgroup analysis shows that the countries’ contribution is highest in Tanzania at 85.23% and Kenya at 80.23%. The types of substance use with a significant impact were combined substance/drugs (89.09%), heroin (80.71%), and alcohol (46.75%). Although alcohol was significantly abused across the region, there is a growing public health impact of other substances, specifically heroin and other combined/injectable drugs. The subgroup analysis further reveals a that cohort study design of 66.10% and a cross-sectional group of 58.98% accounts for variation and heterogeneity. Exacerbated by the study size of frequent studies above 1000 sample size of 76.05%, study period of months group estimate of 73.38% and years estimated 68.18%. However, the variation indicates a comprehensive representation of the regional report. However, the variation in definitions of substance use types (e.g., alcohol, khat, marijuana, heroin, cocaine, injected drugs, and combined substances) could have significantly influenced the results of this analysis. Different studies might define substance use categories in various ways, leading to inconsistent classifications that could distort the overall findings.

Nevertheless, our findings are similar to those reported by Gamarel et al. (95), which revealed high rates of alcohol consumption (21.3%), marijuana usage (27.5%), tobacco use (32.9%), and other illicit substance use (22.5%) in clinical settings. Necho et al. (88) show the average prevalence of AUD among Africans living with HIV/AIDS to be 22.03%. The comprehensive review and meta-analysis report of Nduka and Uthman (32) depicts 33.6% of HIV-positive individuals abusing substances, with prescription medications being the most commonly used in Africa. The report of Birungi et al. (27) has also shown alcohol (22/484, 4.3%) and marijuana (10/484, 2.1%) prevalence among people living with HIV in Uganda and a prevalence of 6.6% for substance use among HIV-positive youth attending CTC in Dodoma (96). However, the variance in the prevalence of substance use among the HIV-infected population in the represented nations found in our study may be due to differences in the socioeconomic status of the various countries, cultural differences, the accessibility and availability of alcoholic beverages, the number of studies conducted in developed countries, and the study setting capability of the investigation. The prevalence of substance misuse and usage among people living with HIV presents several obstacles to treatment outcomes and healthcare delivery. Studies have revealed that depression and other mental health conditions are frequently present in drug addicts infected with HIV, and they are independently linked to the advancement of HIV infection (97).

Furthermore, Merlin et al. (98) have indicated that people with HIV infection frequently have substance use and mood issues. According to Edelman et al. (99), substance use disorders, encompassing tobacco, alcohol, and other drugs, are more prevalent among HIV-positive patients compared to non-HIV patients. This trend holds true for individuals of all ages. Moreover, a high number of new HIV infections are associated with substance usage (100). Our epidemiological characteristic finding has mapped and identified areas with higher prevalence rates or specific risk factors in the East African region, which stockholders may engage in tailoring interventions to these specific regions, ensuring resources are allocated where they are most needed. These findings collectively highlight the need for targeted interventions and regular screening for substance use among people living with HIV in the female and young adult populations in Africa. Substance use among this population is associated with various vulnerabilities, including high-risk behaviours, HIV disease progression, mental health problems, and nonadherence to ART (11). The study by Sandfort et al. (87) indicated that substance use is associated with sexual risk practices, other infections, violence, and transactional sex. Their study also established a link between alcohol consumption and risky sexual behaviours among men who have sex with men (MSM) who abuse substances in Africa.

### Policymaker and future research engagement in the East African region and African continent on substance use among people living with HIV

There is evidence that serious substance use problems increase the death rate among HIV-positive individuals in Africa and act as a stimulator to the unending spread of the disease because substance use has a substantial impact on HIV care outcomes since it can cause noncompliance with ART and have a negative clinical outcome for those living with HIV. For effective intervention against a variety of risk behaviours among substance abusers, it is imperative to comprehend the connection between substance use, neurocognitive impairment, and HIV risk factors. Addressing the complicated overlap between substance use and noncompliance with HIV treatment, as well as frequent mental health issues in this population, integrated treatment for substance abusers with HIV is desperately needed. To increase adherence to ART and optimise treatment outcomes for HIV infection, it is imperative to integrate medical and substance misuse therapies for HIV-positive individuals with substance use difficulties.

Interventions aimed at drug use and sexual risk behaviour are rare, and HIV risk-reduction programs are not widely available in substance use facilities in low-income African nations (101). Prior research indicated that drug use dramatically lowers access to ART, adherence to treatment, and viral resistance in HIV-positive individuals. Thus, it is critical to use epidemiological data to guide policies for managing and preventing substance addiction among individuals living with HIV. This may entail enhancing readily available harm reduction initiatives, such as needle exchange programs and therapy for opioid substitution, to lessen the spread of HIV. It is critical to address substance use and dependence in patients living with HIV infection because research indicates that patients are less likely to receive appropriate HIV care and treatment, including access to ART if these issues are not addressed (102). Healthcare professionals and legislators can gain a better understanding of how substance abuse affects HIV transmission and progression by employing epidemiological data as evidence-based policymaking for substance use prevention and management. Future studies may focus on evaluating the implementation of recreational drug use screening policies and mental health disorders among people living with HIV. In addition, the impact of global economic and political forces on HIV epidemics may improve prevention strategies.

Therefore, this study recommends a holistic approach such as integrating substance use treatment into HIV care programs by combining HIV care with substance use treatment in the study region. By integrating these services, healthcare providers can offer comprehensive care that addresses both HIV management and substance use simultaneously, improving outcomes, reducing stigma, and enhancing overall patient support. Secondly, this study proposes enhancing harm reduction services, a policy that suggests expanding harm reduction strategies, such as needle exchange programs, supervised consumption spaces, and access to naloxone for overdoses. By focusing on reducing the negative health effects of substance use rather than criminalizing it, harm reduction services help prevent the spread of HIV and other infections, reduce overdose deaths, and encourage individuals to seek further treatment or support when ready.

### Limitations and strengthens

The major limitation of this study is that certain studies were not qualified for review, and some were not included in the prevalence estimates because they did not include either the total tested population or cases, a particular age and sex, or the study period. Again, the absence of consistent or precise data makes it difficult to combine research from many nations and evaluate trends over time. Furthermore, only English-language studies were included in our analysis. Additionally, this study focuses on substance use among the HIV-positive population, which informs our search strategy that primarily centred on HIV, hence overlooking several papers on HIV and hepatitis or other infectious diseases of important significance associated with drug misuse. Moreover, most included studies have a gender imbalance in their sample. This imbalance may primarily be responsible for the high prevalence among women in Uganda relative to the worldwide distribution. However, it is also reasonable to surmise that women’s underrepresentation in treatment facilities and the failure of studies to include women may play a part. Lastly, there is a limitation in generalizability of these findings among the countries in this region due to the difference in culture and demographic groups within the East African community.

## Conclusion

In conclusion, the high prevalence and distribution of substance use is a growing concern of public health implications in the studied region. There is a clear need for further research on the prevention/management of the epidemiological distribution of substance use among people living with HIV in East Africa. Regular surveillance surveys and reviews of medical records data may enable the development of targeted interventions. Furthermore, mental health protocol review engagement informs the stakeholders and policymakers of the identification of risk factors and the targeting of specific high-risk groups to improve prevention strategies.

## Data Availability

The original contributions presented in the study are included in the article/**Supplementary Material**. Further inquiries can be directed to the corresponding author.
