# Supplementary material for: Prevalence and epidemiological distribution of substance use among people living with HIV in the East African region: a meta-analysis
Source: Front Psychiatry. 2025 Sep 3;16:1494469. doi: 10.3389/fpsyt.2025.1494469 (PMC12441044; doi:10.3389/fpsyt.2025.1494469)
Supplement: Supplementary file 1 [file DataSheet1.pdf]

Supplementary Table 1. The Newcastle-Ottawa Scale (NOS) quality assessment of the included studies

**Reference**	**Country/region**	**Age**	**Types of substance abuse**	**Sample size**	**Study Period**	**Study type**	**Epidemiological prevalence/Incident**	**Comparability of cohorts**	**Score**
(Birungi et al., 2021)	*	*	*	*	NA	*	*	NA	6
(Swahn et al., 2021)	*	*	*	*	*	*	*	NA	7
(Papas et al., 2021)	*	*	*	*	*	*	*	NA	7
(Likindikoki et al., 2020)	*	*	*	*	*	*	*	NA	7
(Kiene et al., 2019)	*	NA	*	*	*	*	*	NA	6
(Sileo et al., 2019)	*	*	*	*	*	*	*	NA	7
(Leyna et al., 2019)	*	*	*	*	NA	*	*	NA	6
(Hassan et al., 2019)	*	*	*	*	*	*	*	NA	7
(Parcesepe et al., 2019)	*	*	*	*	*	*	*	NA	7
(Duko et al., 2019)	*	*	*	*	NA	*	*	NA	6
(Vancampfort et al., 2019)	*	*	*	*	NA	*	*	NA	6
(Logie et al., 2022)	*	*	*	*	*	*	*	NA	7
(Chang et al., 2022)	*	*	*	*	*	*	*	NA	7
(Miller et al., 2022)	*	*	*	*	NA	*	*	NA	6
(Nyongesa et al., 2021)	*	*	*	*	*	*	*	NA	7
(Kekibiina et al., 2021)	*	*	*	*	*	*	*	NA	7

(Long et al., 2020)	*	*	*	*	*	*	*	NA	7
(Budambula et al., 2018)	*	NA	*	*	*	*	*	NA	6
(Demissie et al., 2018)	*	*	*	*	*	*	*	NA	7
(Hahn et al., 2018)	*	*	*	*	*	*	*	NA	7
(Bultum et al., 2018)	*	*	*	*	*	*	*	NA	7
(Woolf-king et al., 2018)	*	NA	*	*	NA	*	*	NA	5
(Lifson et al., 2017a)	*	*	*	*	NA	*	*	NA	6
(Lifson et al., 2017b)	*	*	*	*	*	*	*	NA	7
(Mmbaga et al., 2017)	*	*	*	*	*	*	*	NA	7
(Kiene et al., 2017)	*	*	*	*	NA	*	*	NA	6
(Guise et al., 2016)	*	NA	*	*	*	*	*	NA	6
(Weiss et al., 2016)	*	*	*	*	*	*	*	NA	7
(Secor et al., 2015)	*	*	*	*	*	*	*	NA	7
(Muturi, 2015)	*	*	*	*	NA	*	*	NA	6
(Kurth et al., 2015)	*	*	*	*	*	*	*	NA	7
(Guise et al., 2015)	*	*	*	*	*	*	*	NA	7
(Rhodes et al., 2015)	*	*	*	*	*	*	*	NA	7
(Tun et al., 2015)	*	*	*	*	*	*	*	NA	7
(Syvertsen et al., 2015)	*	*	*	*	*	*	*	NA	7
(Mwatelah et al., 2015)	*	*	*	*	NA	*	*	NA	6

(Tan et al., 2015)	*	NA	*	*	*	*	*	NA	6
(Soboka et al., 2015)	*	*	*	*	*	*	*	NA	7
(Tran et al., 2015)	*	*	*	*	*	*	*	NA	7
(Sundararajan et al., 2015)	*	*	*	*	NA	*	*	NA	6
(Medley et al., 2014)	*	*	*	*	*	*	*	NA	7
(Soboka et al., 2014)	*	*	*	*	*	*	*	NA	7
(Kruse et al., 2014)	*	*	*	*	NA	*	*	NA	6
(Osman et al., 2013)	*	*	*	*	*	*	*	NA	7
(Bajunirwe et al., 2013)	*	*	*	*	*	*	*	NA	7
(Johnston et al., 2010)	*	*	*	*	*	*	*	NA	7
(Tegang et al., 2010)	*	*	*	*	*	*	*	NA	7
(Williams et al., 2009)	*	*	*	*	*	*	*	NA	7
(Fisher et al., 2008)	*	*	*	*	*	*	*	NA	7
(Ross et al., 2008)	*	*	*	*	*	*	*	NA	7
(Williams et al., 2007)	*	*	*	*	*	*	*	NA	7
(Mccuray et al., 2005)	*	NA	*	*	*	*	*	NA	6
(Shaffer et al., 2004)	*	*	*	*	NA	*	*	NA	6

NA = Non available

Supplementary Table 2. All the inclusive studies on the East Africa regional prevalence of substance abuse/misuse among people living with HIV.

Authors	P/year	doi	title
(Birungi et al., 2021)	2021	10.1080/09540121.2020.1717419	SUBSTANCE USE AMONG HIVINFECTED ADOLESCENTS IN UGANDA RATES AND ASSOCIATION WITH POTENTIAL RISKS AND OUTCOME FACTORS
(Swahn et al., 2021)	2021	10.1007/S10461-021-03301-0	THE INTERSECTION OF ALCOHOL USE GENDER BASED VIOLENCE AND HIV EMPIRICAL FINDINGS AMONG DISADVANTAGED SERVICESEEKING YOUTH IN KAMPALA UGANDA
(Papas et al., 2021)	2021	10.1111/ADD.15112	A RANDOMIZED CLINICAL TRIAL OF A GROUP COGNITIVEBEHAVIORAL THERAPY TO REDUCE ALCOHOL USE AMONG HUMAN IMMUNODEFICIENCY VIRUSINFECTED OUTPATIENTS IN WESTERN KENYA
(Likindikoki et al., 2020)	2020	10.1186/S12954-020-00364-5	PREVALENCE AND RISK FACTORS ASSOCIATED WITH HIV1 INFECTION AMONG PEOPLE WHO INJECT DRUGS IN DAR ES SALAAM TANZANIA A SIGN OF SUCCESSFUL INTERVENTION
(Kiene et al., 2019)	2019	10.1080/09540121.2018.1497135	HAZARDOUS ALCOHOL CONSUMPTION AND ALCOHOLRELATED PROBLEMS ARE ASSOCIATED WITH UNKNOWN AND HIVPOSITIVE STATUS IN FISHING COMMUNITIES IN UGANDA
(Sileo et al., 2019)	2019	10.1371/JOURNAL.PONE.0216892	SUBSTANCE USE AND ITS EFFECT ON ANTIRETROVIRAL TREATMENT ADHERENCE AMONG MALE FISHERFOLK LIVING WITH HIVAIDS IN UGANDA
(Leyna et al., 2019)	2019	10.1186/S12954-019-0346-Y	HIVHCV COINFECTION AND ASSOCIATED RISK FACTORS AMONG INJECTING DRUG USERS IN DAR ES SALAAM TANZANIA POTENTIAL FOR HCV ELIMINATION
(Hassan et al., 2019)	2019	10.3390/IJERPH16050728	EVALUATING THE INTEGRATED METHADONE AND ANTIRETROVIRAL THERAPY STRATEGY IN TANZANIA USING THE REAIM FRAMEWORK
(Parcesepe et al., 2019)	2019	10.1007/S10461-018-2340-X	GENDER DIFFERENCES AND PSYCHOSOCIAL FACTORS ASSOCIATED WITH PROBLEM DRINKING AMONG ADULTS ENROLLING IN HIV CARE IN TANZANIA
(Duko et al., 2019)	2019	10.1186/S13011-019-0212-7	ALCOHOL USE DISORDER AND ASSOCIATED FACTORS AMONG INDIVIDUALS LIVING WITH HIV IN HAWASSA CITY ETHIOPIA A FACILITY BASED CROSS SECTIONAL STUDY
(Vancampfort et al., 2019)	2019	10.1177/0956462419863924	ASSOCIATIONS BETWEEN PHYSICAL INACTIVITY MAJOR DEPRESSIVE DISORDER AND ALCOHOL USE DISORDER IN PEOPLE LIVING WITH HIV IN A UGANDAN FISHING COMMUNITY
(Logie et al., 2022)	2022	10.1136/BMJGH-2021-006583	EXAMINING THE SUBSTANCE USE VIOLENCE AND HIV AND AIDS SAVA SYNDEMIC AMONG URBAN REFUGEE YOUTH IN KAMPALA UGANDA CROSSSECTIONAL SURVEY FINDINGS
(Chang et al., 2022)	2022	10.1002/JIA2.26029	HAZARDOUS ALCOHOL USE AND HIV INDICATORS IN SIX AFRICAN COUNTRIES RESULTS FROM THE POPULATIONBASED HIV IMPACT ASSESSMENTS 20152017
(Miller et al., 2022)	2022	10.1186/S12889-022-14295-2	UNHEALTHY ALCOHOL USE AND INTIMATE PARTNER VIOLENCE AMONG MEN AND WOMEN LIVING WITH HIV IN UGANDA
(Nyongesa et al., 2021)	2021	10.1186/S13011-021-00422-6	ALCOHOL AND ILLICIT DRUG USE AMONG YOUNG PEOPLE LIVING WITH HIV COMPARED TO THEIR UNINFECTED PEERS FROM THE KENYAN COAST PREVALENCE AND RISK INDICATORS
(Kekibiina et al., 2021)	2021	10.1186/S12888-021-03464-Z	POSTTRAUMATIC STRESS DISORDER AMONG PERSONS WITH HIV WHO ENGAGE IN HEAVY ALCOHOL CONSUMPTION IN SOUTHWESTERN UGANDA
(Long et al., 2020)	2020	10.1371/JOURNAL.PONE.0242817	ALCOHOL USE AND VIRAL SUPPRESSION IN HIVPOSITIVE KENYAN FEMALE SEX WORKERS ON ANTIRETROVIRAL THERAPY
(Budambula et al., 2018)	2018	10.1186/S12889-018-5100-Y	SOCIODEMOGRAPHIC AND SEXUAL PRACTICES ASSOCIATED WITH HIV INFECTION IN KENYAN INJECTION AND NONINJECTION DRUG USERS
(Demissie et al., 2018)	2018	10.2989/16085906.2018.1511604	PREVALENCE OF HIV AND OTHER INFECTIONS AND INJECTION BEHAVIOURS AMONG PEOPLE WHO INJECT DRUGS IN ADDIS ABABA ETHIOPIA
(Hahn et al., 2018)	2018	10.1097/QAI.0000000000001624	ALCOHOL USE AND HIV DISEASE PROGRESSION IN AN ANTIRETROVIRAL NAIVE COHORT

(Bultum et al., 2018)	2018	10.1371/JOURNAL.PONE.0189312	ALCOHOL USE DISORDER AND ASSOCIATED FACTORS AMONG HUMAN IMMUNODEFICIENCY VIRUS INFECTED PATIENTS ATTENDING ANTIRETROVIRAL THERAPY CLINIC AT BISHOFTU GENERAL HOSPITAL OROMIYA REGION ETHIOPIA
(Woolf-King et al., 2018)	2018	10.1007/s10508-017-1131-1	ALCOHOL USE AND UNPROTECTED SEX AMONG HIVINFECTED UGANDAN ADULTS FINDINGS FROM AN EVENTLEVEL STUDY
(Lifson et al., 2017a)	2017	10.1089/AID.2016.0274	FREQUENT USE OF KHAT AN AMPHETAMINELIKE SUBSTANCE AS A RISK FACTOR FOR POOR ADHERENCE AND LOST TO FOLLOWUP AMONG PATIENTS NEW TO HIV CARE IN ETHIOPIA
(Lifson et al., 2017b)	2017	10.1186/S13722-016-0069-2	PREVALENCE AND FACTORS ASSOCIATED WITH USE OF KHAT A SURVEY OF PATIENTS ENTERING HIV TREATMENT PROGRAMS IN ETHIOPIA
(Mmbaga et al., 2017)	2017	10.1097/OLQ.0000000000000555	PREVALENCE AND PREDICTORS OF HUMAN IMMUNODEFICIENCY VIRUS AND SELECTED SEXUALLY TRANSMITTED INFECTIONS AMONG PEOPLE WHO INJECT DRUGS IN DAR ES SALAAM TANZANIA A NEW FOCUS TO GET TO ZERO
(Kiene et al., 2017)	2017	10.1186/s12879-016-2162-2	DEPRESSION ALCOHOL USE AND INTIMATE PARTNER VIOLENCE AMONG OUTPATIENTS IN RURAL UGANDA VULNERABILITIES FOR HIV STIS AND HIGH RISK SEXUAL BEHAVIOR
(Guise et al., 2016)	2016	10.1080/09540121.2016.1191606	ACCESS TO HIV TREATMENT AND CARE FOR PEOPLE WHO INJECT DRUGS IN KENYA A SHORT REPORT
(Weiss et al., 2016)	2016	10.3390/IJERPH13020153	HIGH LEVELS OF PERSISTENT PROBLEM DRINKING IN WOMEN AT HIGH RISK FOR HIV IN KAMPALA UGANDA A PROSPECTIVE COHORT STUDY
(Secor et al., 2015)	2015	10.1097/QAD.0000000000000846	DEPRESSION SUBSTANCE ABUSE AND STIGMA AMONG MEN WHO HAVE SEX WITH MEN IN COASTAL KENYA
(Muturi, 2015)	2015	10.2989/16085906.2015.1016986	GENDER AND HIV INFECTION IN THE CONTEXT OF ALCOHOLISM IN KENYA
(Kurth et al., 2015)	2015	10.1097/QAI.0000000000000769	HIV PREVALENCE ESTIMATED INCIDENCE AND RISK BEHAVIORS AMONG PEOPLE WHO INJECT DRUGS IN KENYA
(Guise et al., 2015)	2015	10.1186/S12954-015-0061-2	A QUALITATIVE ANALYSIS OF TRANSITIONS TO HEROIN INJECTION IN KENYA IMPLICATIONS FOR HIV PREVENTION AND HARM REDUCTION
(Rhodes et al., 2015)	2015	10.1136/BMJOPEN-2014-007198	IS THE PROMISE OF METHADONE KENYAS SOLUTION TO MANAGING HIV AND ADDICTION A MIXEDMETHOD MATHEMATICAL MODELLING AND QUALITATIVE STUDY
(Tun et al., 2015)	2015	10.1007/S10461-014-0936-3	HIV AND STI PREVALENCE AND INJECTION BEHAVIORS AMONG PEOPLE WHO INJECT DRUGS IN NAIROBI RESULTS FROM A 2011 BIOBEHAVIORAL STUDY USING RESPONDENTDRIVEN SAMPLING
(Syvertsen et al., 2015)	2015	S0376-8716(15)00141-6	EVIDENCE OF INJECTION DRUG USE IN KISUMU KENYA IMPLICATIONS FOR HIV PREVENTION
(Mwatelah et al., 2015)	2015	10.1371/JOURNAL.PONE.0132287	COINFECTION BURDEN OF HEPATITIS C VIRUS AND HUMAN IMMUNODEFICIENCY VIRUS AMONG INJECTING HEROIN USERS AT THE KENYAN COAST
(Tan et al., 2015)	2015	10.1371/JOURNAL.PONE.0145578	EPIDEMIOLOGY OF DRUG USE AND HIVRELATED RISK BEHAVIORS AMONG PEOPLE WHO INJECT DRUGS IN MWANZA TANZANIA
(Soboka et al., 2015)	2015	10.1186/S12888-015-0446-5	KHAT USE IN PEOPLE LIVING WITH HIV A FACILITYBASED CROSSSECTIONAL SURVEY FROM SOUTH WEST ETHIOPIA
(Tran et al., 2015)	2015	10.1097/QAI.0000000000000582	IMPLEMENTATION AND OPERATIONAL RESEARCH LINKAGE TO CARE AMONG METHADONE CLIENTS LIVING WITH HIV IN DAR ES SALAAM TANZANIA
(Sundararajan et al., 2015)	2015	10.1007/S10461-014-0918-5	QUALITATIVE STUDY OF CHANGES IN ALCOHOL USE AMONG HIVINFECTED ADULTS ENTERING CARE AND TREATMENT FOR HIVAIDS IN RURAL SOUTHWEST UGANDA
(Medley et al., 2014)	2014	10.1080/09540121.2014.911809	ALCOHOL USE AND ITS ASSOCIATION WITH HIV RISK BEHAVIORS AMONG A COHORT OF PATIENTS ATTENDING HIV CLINICAL CARE IN TANZANIA KENYA AND NAMIBIA
(Soboka et al., 2014)	2014	10.1186/1756-0500-7-828	ALCOHOL USE DISORDERS AND ASSOCIATED FACTORS AMONG PEOPLE LIVING WITH HIV WHO ARE ATTENDING SERVICES IN SOUTH WEST ETHIOPIA
(Kruse et al., 2014)	2014	10.1007/S10461-014-0737-8	TOBACCO USE AMONG ADULTS INITIATING TREATMENT FOR HIV INFECTION IN RURAL UGANDA
(Osman et al., 2013)	2013	10.1089/AID.2012.0182	DIVERSITY OF HIV TYPE 1 AND DRUG RESISTANCE MUTATIONS AMONG INJECTING DRUG USERS IN KENYA
(Bajunirwe et al., 2013)	2013	10.1186/1471-2458-13-430	ALCOHOL USE AND HIV SEROSTATUS OF PARTNER PREDICT HIGHRISK SEXUAL BEHAVIOR AMONG PATIENTS RECEIVING ANTIRETROVIRAL THERAPY IN SOUTH WESTERN UGANDA

(Johnston et al., 2010)	2010	10.1016/J.DRUGPO.2010.06.001	HIV RISK AND THE OVERLAP OF INJECTING DRUG USE AND HIGHRISK SEXUAL BEHAVIOURS AMONG MEN WHO HAVE SEX WITH MEN IN ZANZIBAR UNGUJA TANZANIA
(Tegang et al., 2010)	2010	10.1080/17290376.2010.9724972	CONCURRENT SEXUAL AND SUBSTANCEUSE RISK BEHAVIOURS AMONG FEMALE SEX WORKERS IN KENYAS COAST PROVINCE FINDINGS FROM A BEHAVIOURAL MONITORING SURVEY
(Williams et al., 2009)	2009	10.1521/AEAP.2009.21.5.474	HIV SEROPREVALENCE IN A SAMPLE OF TANZANIAN INTRAVENOUS DRUG USERS
(Fisher et al., 2008)	2008	10.1097/OLQ.0B013E3181677547	PATTERNS OF ALCOHOL USE PROBLEM DRINKING AND HIV INFECTION AMONG HIGHRISK AFRICAN WOMEN
(Ross et al., 2008)	2008	10.4269/ajtmh.2008.79.338	DRUG USE CAREERS AND BLOODBORNE PATHOGEN RISK BEHAVIOR IN MALE AND FEMALE TANZANIAN HEROIN INJECTORS
	2007	10.1007/s10461-006-9102-x	DIFFERENCES IN HIV RISK BEHAVIORS BY GENDER IN A SAMPLE OF TANZANIAN INJECTION DRUG USERS
	2005	#N/A	HEROIN AND HIV RISK IN DAR ES SALAAM TANZANIA YOUTH HANGOUTS MAGETO AND INJECTING PRACTICES
(Shaffer et al., 2004)	2004	#N/A	ALCOHOL ABUSE AMONG PATIENTS WITH AND WITHOUT HIV INFECTION ATTENDING PUBLIC CLINICS IN WESTERN KENYA

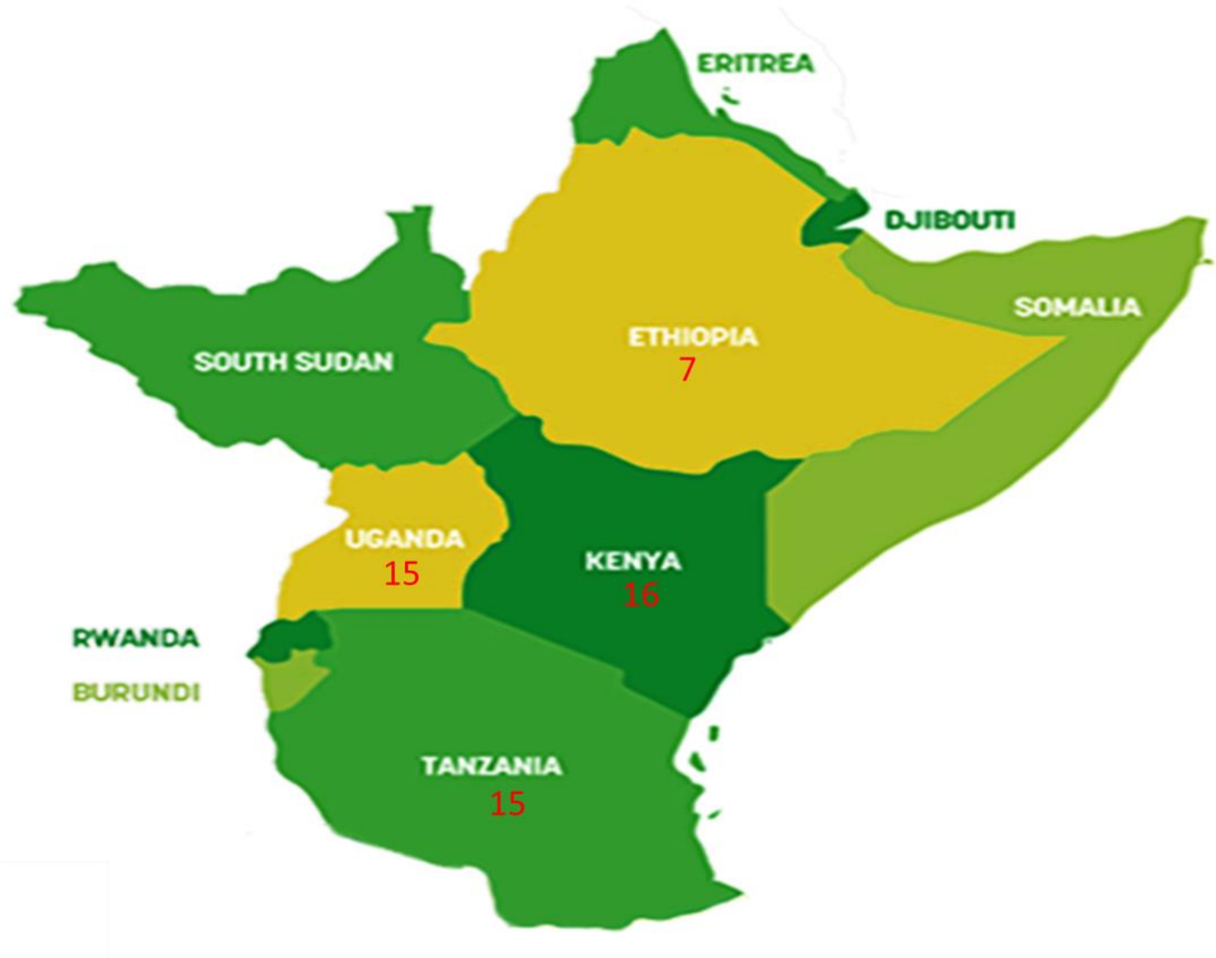

Supplementary Figure 1. Selected studies hotspot on the East Africa regional prevalence of substance abuse/misuse among people living with HIV.

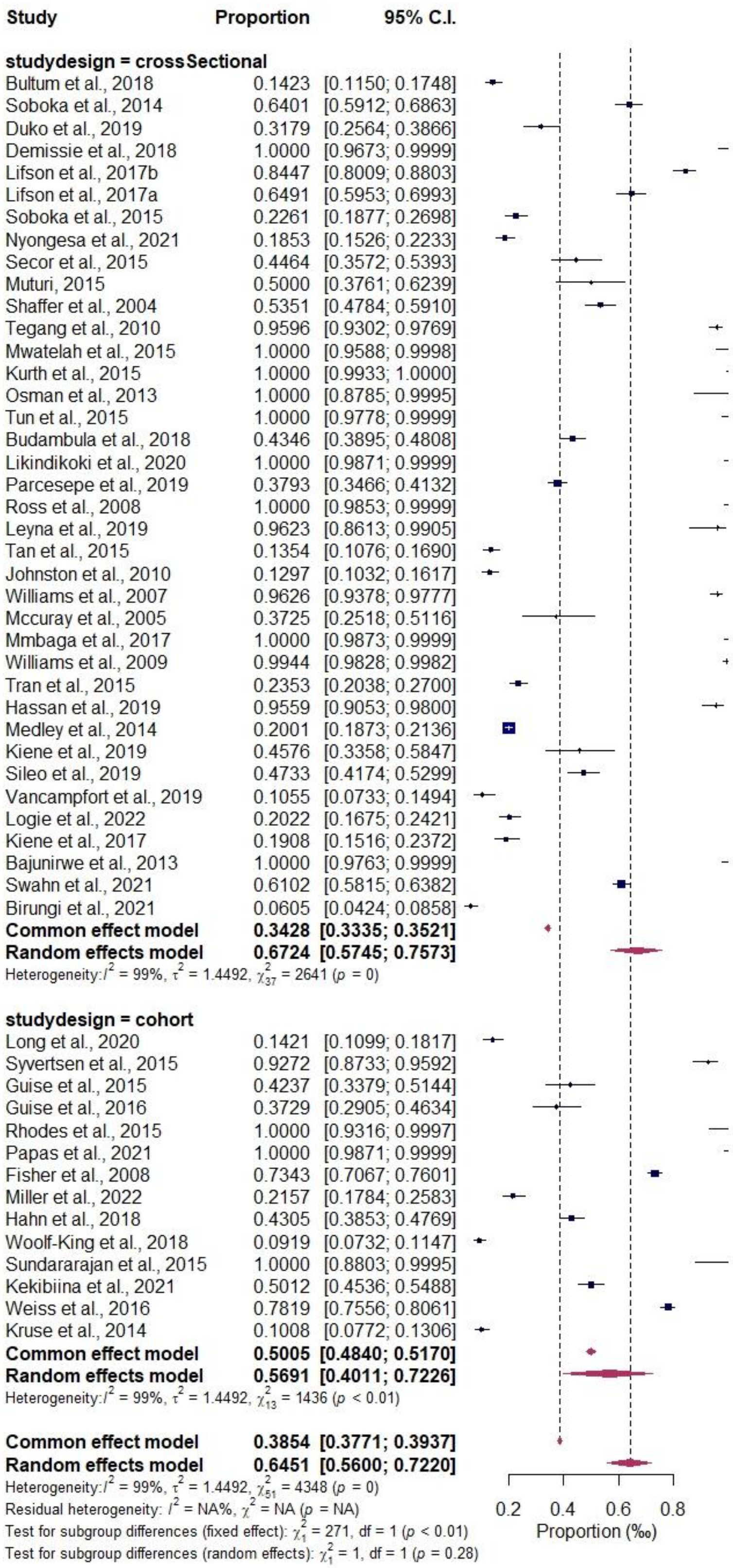

Supplementary Figure 2. Forest plot of the subgroup analysis by study design

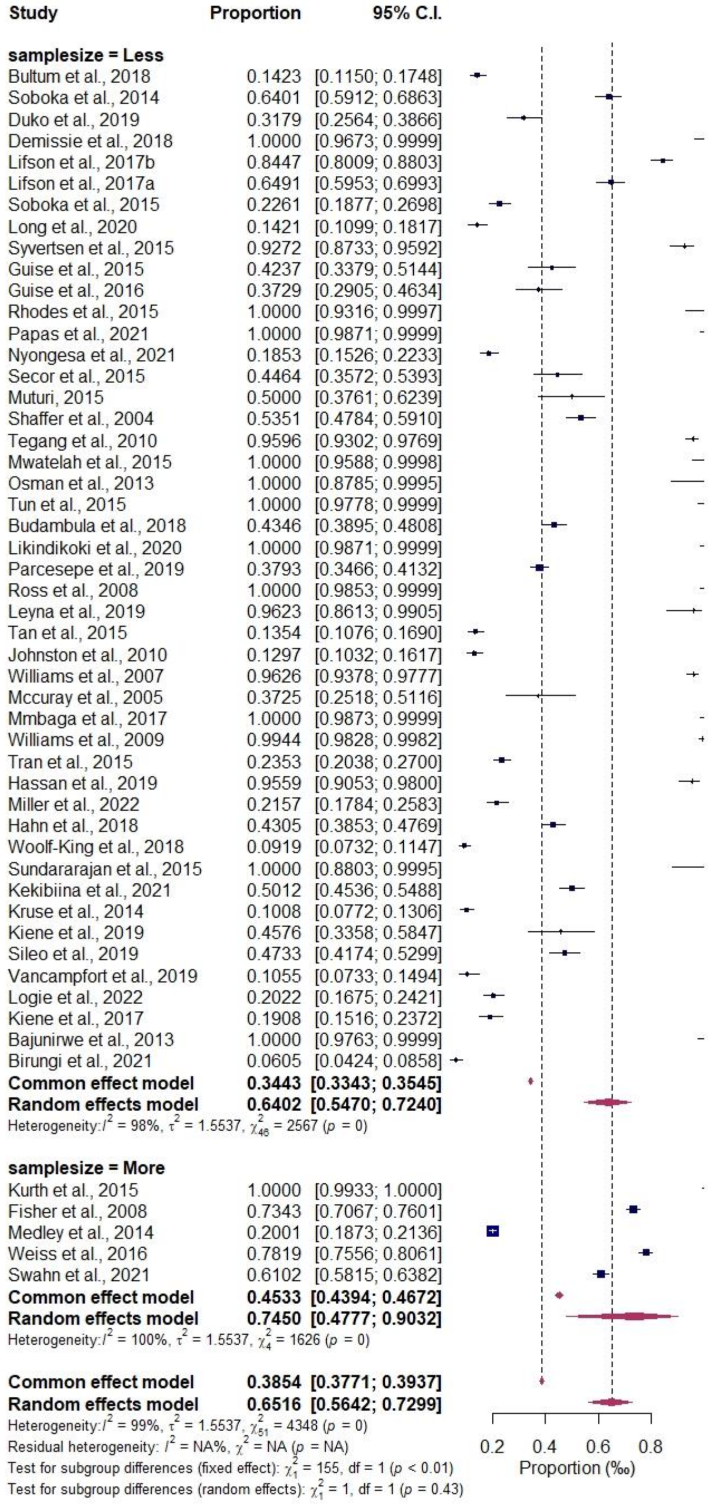

Supplementary Figure 3. Forest plot of the subgroup analysis by sample size

Table 2 Pooled estimates of Types substance abuse/misuse and East African regional epidemiological characteristics.

				Random effect model		
	Studies	Estimate effect	95% CI	prevalence (%)	95% CI (%)	Heterogeneity (I^2)
Types substance abuse/misuse						
Alcohol	25	-0.0576	-0.5804;0.4856	48.56	35.89;61.42	99%
combined **	7	1.7126	0.5515;0.8396	83.96	64.98;93.65	99%
Heroin *	8	1.1521	0.0479;0.7492	74.92	53.04;88.77	98%
Injected Drugs ***	6	2.8913	1.5633;0.9445	94.45	83.38;98.29	97%
Khart	2	-0.2499	-2.0879;0.4237	42.37	11.21;81.07	99%
Methadone *	3	1.7639	0.0598;0.8464	84.64	52.11;96.54	98%
Tobacco		-2.1307	-4.6831;0.1008	10.08	0.91;57.69	-
Signif. codes: 0 ‘***’ 0.001 ‘**’ 0.01 ‘*’ Vs Alcohol						
East African Nations						
Ethiopia	7	0.305	-0.713;0.5757	57.57	32.89;78.97	99%
Kenya [a]	16	1.1619	-0.0892;0.8126	81.26	67.69;89.97	97%
Tanzania*	13	1.3358	0.0506;0.8376	83.76	70.19;91.87	99%
Tanzania & other nations	1	-1.6906	-4.4618;0.2001	20.01	1.86;76.71	-
Uganda	15	-0.8051	-2.0372;0.3775	37.75	23.25;54.84	99%
Signif. codes: 0.01 ‘*’ 0.05 ‘[a]’ Vs Ethiopia						
